# Mechanisms Governing DDK Regulation of the Initiation of DNA Replication

**DOI:** 10.3390/genes8010003

**Published:** 2016-12-22

**Authors:** Larasati, Bernard P. Duncker

**Affiliations:** Department of Biology, University of Waterloo, 200 University Avenue West, Waterloo, ON N2L3G1, Canada; llarasat@uwaterloo.ca

**Keywords:** DNA replication, DDK, Dbf4, Cdc7, MCM, Rad53, cell cycle checkpoint, Rif1, Sld3

## Abstract

The budding yeast Dbf4-dependent kinase (DDK) complex—comprised of cell division cycle (Cdc7) kinase and its regulatory subunit dumbbell former 4 (Dbf4)—is required to trigger the initiation of DNA replication through the phosphorylation of multiple minichromosome maintenance complex subunits 2-7 (Mcm2-7). DDK is also a target of the radiation sensitive 53 (Rad53) checkpoint kinase in response to replication stress. Numerous investigations have determined mechanistic details, including the regions of Mcm2, Mcm4, and Mcm6 phosphorylated by DDK, and a number of DDK docking sites. Similarly, the way in which the Rad53 forkhead-associated 1 (FHA1) domain binds to DDK—involving both canonical and non-canonical interactions—has been elucidated. Recent work has revealed mutual promotion of DDK and synthetic lethal with *dpb11-1* 3 (Sld3) roles. While DDK phosphorylation of Mcm2-7 subunits facilitates their interaction with Sld3 at origins, Sld3 in turn stimulates DDK phosphorylation of Mcm2. Details of a mutually antagonistic relationship between DDK and Rap1-interacting factor 1 (Rif1) have also recently come to light. While Rif1 is able to reverse DDK-mediated Mcm2-7 complex phosphorylation by targeting the protein phosphatase glycogen 7 (Glc7) to origins, there is evidence to suggest that DDK can counteract this activity by binding to and phosphorylating Rif1.

## 1. Introduction

In an unperturbed cell cycle, budding yeast Dbf4-dependent kinase (DDK) complex triggers the initiation of DNA replication mainly through the phosphorylation of minichromosome maintenance complex subunits 2-7 (Mcm2-7) (reviewed in [1]). When DNA damage or dNTP depletion results in checkpoint activation, the normal role of DDK is opposed by radiation sensitive 53 (Rad53) kinase, which phosphorylates DDK, leading to its dissociation from chromatin [2,3,4,5,6]. Recently, a much better understanding of the way in which DDK associates with both Mcm2-7 and Rad53 (structurally and functionally) has been gained. This review will focus on genetic and molecular studies that have identified and characterized the subunits of Mcm2-7 which mediate the binding of DDK, and those that are the critical targets of DDK phosphorylation. Similarly, crucial mechanistic details of both canonical and non-canonical ways in which the Rad53 forkhead-associated 1 domain (FHA1) interacts with DDK have been determined. Recently, roles for additional protein factors in regulating DDK stimulation have also been uncovered. These include synthetic lethal with *dpb11-1* 3 (Sld3), which both stimulates DDK phosphorylation of Mcm2 and binds to DDK-phosphorylated Mcm4 and Mcm6; and Rap1-interacting factor 1 (Rif1), which counteracts DDK activity by recruiting the protein phosphatase glycogen 7 (Glc7) to dephosphorylate Mcm4. Finally, evidence supporting a role for DDK in coordinating the initiation of DNA replication with sister chromatid cohesion will be discussed.

## 2. Insights into DDK Interactions with Mcm2-7

**Figure 1:**
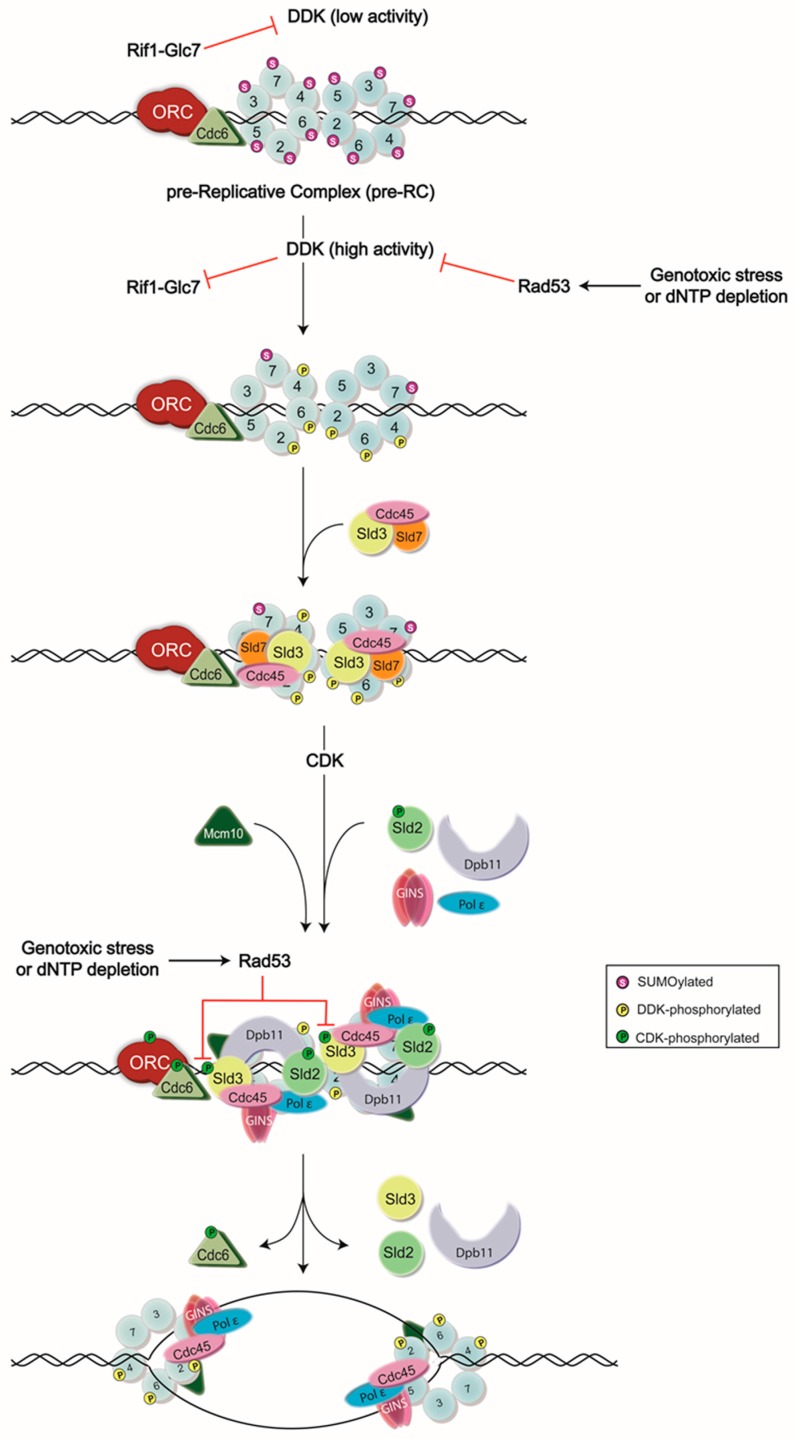
Model of DNA replication initiation. DNA replication is initiated by the assembly of the pre-Replicative Complex (pre-RC) at G1 phase, which is then followed by a series of phosphorylation events carried out by Dbf4-dependent kinase (DDK) and cyclin-dependent kinase (CDK) to generate the active form of the CMG (Cdc45-Mcm2-7-go-ichi-ni-san(GINS)) helicase. Normally, DDK activity is low until the end of G1 phase, as Dbf4—the regulatory subunit of DDK—is degraded in an anaphase promoting complex (APC)-dependent manner [12,13,14,15]. However, some Dbf4 that has escaped this process can provide residual DDK activity, contributing to potential premature Mcm2-7 complex phosphorylation. To avoid this, Rif1 recruits the protein phosphatase Glc7 to dephosphorylate the DDK targets. High activity of DDK in late G1 phase is proposed as a mechanism to inhibit Rif1-Glc7 activity [16,17]. DDK activity is also inhibited by the S-phase checkpoint kinase, Rad53, during exposure to genotoxic agents or dNTP depletion. Rad53 binds to and phosphorylates Dbf4 to remove DDK from chromatin and prevent subsequent origin firing [2,3,4,5,6]. Rad53 also phosphorylates an essential target of CDK, Sld3, to ensure the inhibition of DNA replication during replication stress [3,4], ORC: Origin Recognition Complex.

One of the essential players in the initiation of eukaryotic DNA replication is the DDK complex, comprised of the serine-threonine kinase cell division cycle 7 (Cdc7), and its regulatory subunit, dumbbell former 4 (Dbf4). In the budding yeast *Saccharomyces cerevisiae*, each protein is encoded by a single gene, and deletion of either in a haploid strain is lethal [7]. Recently developed in vitro systems which recapitulate the molecular events culminating in origin firing have further demonstrated that the inclusion of DDK is absolutely required for the initiation of DNA replication [8,9,10,11]. The crucial function of DDK is to phosphorylate the Mcm2-7 ring, part of the larger CMG (Cdc45-Mcm2-7-go-ichi-ni-san (GINS)) replicative DNA helicase complex formed at origins of DNA replication (reviewed in [1]). The onset of these events is triggered by a rise in Dbf4 levels at the end of G1-phase, which fall after mitosis as Dbf4 is degraded in an anaphase promoting complex (APC)-dependent manner [12,13,14,15]. The high levels of active DDK at the end of G1-phase are also important for overcoming Rif1-Glc7 activity (discussed below) [16,17]. In recent years, a much higher-resolution understanding of these mechanisms has been obtained (summarized in Figure 1).

It has been known for some time that DDK is essential for DNA replication in vivo, likely due to its phosphorylation of Mcm2-7 inducing a conformational change, thereby favoring interaction with other firing factors. A P83L mutation in Mcm5 encoded by the *mcm5-bob1* allele can bypass DDK’s requirement for viability, presumably mimicking a conformational change that facilitates DNA replication [18,19]. Similarly, some initial insight as to which residues of the Mcm2-7 subunits are the critical DDK targets was provided through a report that pointed to the N-terminal serine/threonine-rich domain (NSD) of Mcm4 as being a target of DDK as well as being required for cell growth and S-phase progression [20]. To test the hypothesis that the NSD is inhibitory to the activation of origins, an allele of *MCM4* lacking the NSD was transformed into temperature-sensitive *cdc7-4* and *dbf4-1* budding yeast strains and—reminiscent of *mcm5-bob1*—found to complement the growth defects at non-permissive temperatures [21]. Further examination of the NSD revealed that it could be functionally divided into overlapping proximal (amino acids 74–174) and distal (amino acids 2–145) regions. The proximal region inhibits origin activation, as demonstrated by a comparison of wild-type *MCM4* and *mcm4∆74-174* strains. When both were exposed to the ribonucleotide reductase inhibitor hydroxyurea (HU, which synchronizes cells in early S-phase by provoking a checkpoint response), the *MCM4* cells only allowed origins that are normally active in early S-phase to fire, whereas with the *mcm4∆74-174* strain, both early- and late-firing origins were activated. In contrast, the distal region was found to restrict the rate of replication fork progression [22,23].

Mcm2 has also been identified as an important DDK target, and is phosphorylated at serines 164 and 170 [24,25,26]. Plasmid-based expression of an allele where sequences encoding the two serines were changed to specify alanines, *mcm2-2A*, acted in a dominant negative fashion in an *MCM2* wild-type strain, resulting in severe growth defects. When the same *mcm2-2A* mutant was expressed at wild-type levels from a plasmid in a temperature-activated degron (td)-tagged *mcm2* strain at 37 °C (a temperature at which the td-tagged Mcm2 is degraded), again severe growth defects were observed, and fluorescence-activated cell sorting (FACS) analysis revealed impaired S-phase progression. Interestingly, in both cases, the defects could be partially suppressed by the *mcm5-bob1* mutation [26]. Mcm2 and Mcm5 lie adjacent to one another in the Mcm2-7 ring, and disruption of the interaction between the two of them leads to an opening, which allows for loading onto double-stranded DNA [27,28,29]. Insight as to the possible biological role of Mcm2 modification by DDK was provided by the observation that DDK-phosphorylated Mcm2 dissociates from Mcm5 and triggers opening of the Mcm2-7 ring [26] to allow extrusion of single-stranded DNA generated from origin melting. Electron microscopy analysis of *Drosophila melanogaster* Mcm2-7 suggests that the Mcm2-Mcm5 gap is later sealed through the interaction of the Mcm2-7 ring with Cdc45 and GINS [30]. As is the case for Mcm2 and Mcm4, Mcm6 has an unstructured N-terminal domain including several DDK target sites [29], and is phosphorylated by this kinase complex in vitro [31,32]. Recently, both DDK-phosphorylated Mcm4 and Mcm6 were shown to bind Sld3, which in turn recruits Cdc45 to origins (discussed below).

In addition to characterizing the regions of MCM subunits that are phosphorylated, insight has been gained regarding the way in which the DDK complex is targeted to Mcm2-7. Sequential analysis of each MCM subunit’s ability to bind the DDK components through both two-hybrid assays and co-immunoprecipitation analysis revealed that Dbf4 and Cdc7 bind to Mcm2 and Mcm4, respectively [33], and DDK docking regions have been uncovered in these two MCM subunits [20,24,33]. In the case of Mcm4, a region comprising amino acids 175–333 was found to mediate binding by DDK [20], while two different regions on Mcm2 are required, including amino acids 2–63 [33] and 204–278 [24]. Interestingly, while structural studies have shown that Mcm2 and Mcm4 are not in close proximity in a single Mcm2-7 hexamer, the situation is different with the double hexameric form known to be loaded onto origins of DNA replication, where these subunits lie adjacent to one another, forming a bipartite DDK binding site, consistent with the finding that the double hexamer is a preferred DDK substrate over the single hexamer [34,35]. Previous work has revealed that DDK interacts with Mcm2 through the conserved Dbf4 N- and C-motifs [36,37], however little is known about the Cdc7 region that interacts with Mcm4.

While Mcm10 does not share sequence homology with Mcm2-7 [38] and is not included in the Mcm2-7 ring, it is nevertheless indispensable for DNA replication [11,38]. A recent study showed that both DDK subunits associate with Mcm10 in vitro, with Dbf4 binding more strongly than Cdc7 [39], which is consistent with an earlier finding that Cdc23 (homolog of Mcm10 in fission yeast *Schizosaccharomyces pombe*) binds to Dfp1 (homolog of Dbf4 in fission yeast) [40]. Mcm10 also interacts with Mcm2-7 [38,41,42,43,44], and the strength of this interaction is increased in the presence of DDK and cyclin-dependent kinase (CDK) [45], which may facilitate double hexamer separation [46]. Moreover, Mcm10 increases DDK phosphorylation of Mcm2 [39,40] and the Mcm2-7 complex as a whole [40] in vitro.

## 3. Regulation of DDK Activity by Rad53

The ability of DDK to phosphorylate MCM subunits can be impeded by the checkpoint kinase Rad53, which is known to bind Dbf4 primarily through its FHA1 domain [47]. Under conditions where DNA is damaged or cellular dNTP pools are depleted, Dbf4 is a target of Rad53, which results in removal of the DDK complex from chromatin [2], thereby inhibiting further origin firing [3,4,5,6]. Furthermore, in vitro phosphorylation of the DDK complex by Rad53 has been found to inhibit the phosphorylation of Mcm2 by DDK [48]. Numerous Rad53 phosphorylation sites have been identified in Dbf4, and mutation of four of these to alanines in a strain for which Rad53 phosphorylation sites in Sld3 were similarly mutated resulted in late origin firing, despite exposure to HU [3]. More recently, characterization of the Dbf4 region required for binding Rad53 revealed that a stretch including amino acid residues 105–221 is both necessary and sufficient for the interaction of Dbf4 and Rad53. A crystal structure was subsequently obtained, confirming a BRCA1 C terminus (BRCT)fold, but with an additional N-terminal alpha-helix required for FHA1 binding, and was therefore designated the H-BRCT domain [49]. As FHA domains are known to bind phosphothreonine-containing motifs, each H-BRCT threonine was systematically mutated, but none of these changes resulted in an abrogation of the interaction with FHA1. Subsequently, a combination of bioinformatics, nuclear magnetic resonance (NMR) spectroscopy, and two-hybrid analysis uncovered a non-canonical lateral surface patch on Rad53 FHA1 that binds to Dbf4 H-BRCT, distinct from its phosphothreonine epitope-binding domain [50]. Importantly, the Rad53 FHA1 domain is able to simultaneously engage Dbf4 H-BRCT and a Cdc7 phosphoepitope known to be recognized by Rad53 [50,51], suggesting a bipartite mode of interaction with the DDK complex. Indeed, this has now been confirmed through the elucidation of the crystal structure of Rad53 FHA1 simultaneously bound to Dbf4 and the phosphorylated Cdc7 peptide [52]. A requirement for FHA1 interaction with both DDK subunits may serve to simultaneously ensure that this only occurs during a checkpoint response (canonical phosphothreonine interaction with Cdc7), and exhibits substrate specificity (non-canonical interaction with Dbf4).

## 4. Mutual Promotion of Sld3 and DDK Activities

Sld3 is a key factor in the initiation of DNA replication and represents an essential target of CDK at this point in the cell cycle [53,54,55]. Sld3 associates with early-firing origins in G1 phase and late-firing origins in late S-phase [56], consistent with it being one of the limiting factors that differentiate early and late origins [55,57]. It binds to both Mcm2-7 and Cdc45, thus serving to recruit the latter to origins [56,58]. In recruiting Cdc45, Sld3 forms a complex with Sld7 [55], which acts to reduce Sld3’s affinity to Cdc45 [59], likely helping Sld3 to dissociate from the origin while Cdc45 remains and eventually moves with the replication fork as a part of the CMG helicase. GINS may also help to displace Sld3 from origins, as they compete with each other for Mcm2-7 binding [58]. Like Dbf4, Sld3 is targeted by Rad53 phosphorylation as a mechanism to inhibit origin firing in response to DNA damage [3,4].

For some time, it has been known that Sld3’s association with origins of DNA replication is DDK-dependent [55,60], but the molecular mechanisms involved have been uncovered more recently. Sld3 binds to Mcm2-7 [58], which facilitates its recruitment to origins. An in vitro replication system comprised of origin DNA attached to magnetic beads supplemented with purified budding yeast replication proteins was used to show that Sld3 binds loaded Mcm2-7 in a manner dependent upon DDK [61]. Further analysis revealed that Sld3 amino acids 510–545 mediate this interaction [61]. Interestingly, this region includes many of the sites that Rad53 phosphorylates to inhibit origin firing [3], and preincubation of Sld3 with Rad53 prevented it from binding MCM in the presence of DDK [61], in much the same way as Rad53 phosphorylation prevents Sld3 from interacting with scaffold protein Dpb11 (see Figure 1) [3]. This same system was further used to examine MCM subunit binding, and revealed that Sld3 specifically interacts with DDK-phosphorylated Mcm4 and Mcm6 [61]. To test whether the binding of Sld3 to Mcm4 and Mcm6 represents the essential function of DDK, Mcm4 and Mcm6 phosphomimic mutants were generated for which N-terminal DDK-targeted serine and threonine residues were substituted with negatively charged aspartate residues (Mcm4-25D, Mcm6-25D), and were able to support roughly 60% the wild-type level of DNA replication in the absence of DDK. Two recent studies have also reported DDK-dependent interactions between Sld3 and Mcm2 through pull-down, co-immunoprecipitation, and two hybrid assays [10,62]. Intriguingly, the crystal structure of Sld3 uncovered two conserved basic patches close to one another with the potential of mediating interactions with phosphorylated Mcm2-7 subunits [63]. One of these (amino acids 301–330) has been found to act as a Cdc45-binding interface [63], while mutation of the second patch for the *sld3-4E* mutant (K188E, R192E, K404E, K405E) resulted in disrupted interactions with Mcm2 and Mcm6, but not Cdc45 [62]. Importantly, a similar mutation of this region (K181E, R186E, R192E, K404E, K405E) also maintained an interaction with Cdc45, but displayed a severe growth inhibition phenotype, and this was mirrored by a failure of *sld3-4E* to support growth in place of the wild-type *SLD3* allele [62,63]. As with Mcm4 and Mcm6, the N-terminus of Mcm2 has proven to be crucial for Sld3 binding, as Mcm2 amino acids 1–390 are sufficient for this interaction, but amino acids 1–299 are not [62]. Confirmation of the physiological importance of Sld3 interactions with the Mcm2 and Mcm6 N-termini, was obtained through in vivo complementation assays, in which deletion mutants with disrupted Sld3 binding for Mcm2 (Δ300–390) or Mcm6 (Δ1–122) failed to support growth or S-phase progression in the absence of wild-type Mcm2 or Mcm6 expression, respectively [62]. Furthermore, quantitative PCR analysis of chromatin immunoprecipitation samples (ChIP-qPCR) revealed that the *mcm6∆1-122* mutant is deficient in recruiting both Sld3 and the single-stranded DNA binding protein replication protein A (RPA) to early-firing origin ARS607, consistent with a defect in replication initiation [62].

Interestingly, there is some evidence to suggest that—in addition to DDK facilitating the association of Sld3 with origins of DNA replication—Sld3 in turn may aid DDK in carrying out one of its roles. As mentioned above, DDK phosphorylates Mcm2 at serines 164 and 170 [24,25,26]. In vitro, the addition of either full-length Sld3 or its C-terminus alone was able to substantially enhance DDK phosphorylation of Mcm2 [64]. Further evidence for the importance of this stimulatory role was obtained by generating a *SLD3* mutant, *sld3-m16* (Sld3-S556A, H557A, S558A, T559A), defective in aiding DDK with Mcm2 phosphorylation, but competent with respect to other functions, including binding to Dpb11, Mcm2-7, Cdc45, and T-rich single-stranded origin DNA. Reminiscent of the *mcm2-2A* mutant, expression of *sld3-m16* resulted in a dominant-negative growth defect phenotype, and a decrease in association between Mcm2-7 and GINS was observed, pointing to a defect in CMG helicase assembly [64]. This mutually stimulatory relationship between DDK and Sld3 activities likely represents an important positive feedback loop that helps push origins past the threshold of CMG formation required for origin firing.

## 5. Opposing Activities of SUMOylation, Rif1, and DDK

Rif1 was initially identified as a regulator of telomeric length [65], but has more recently been implicated in the regulation of DNA replication in budding yeast, fission yeast, and mammalian cells [66,67,68,69,70]. More specifically, several lines of evidence point towards an important role for Rif1 in opposing the MCM phosphorylation activity of DDK. For example, temperature-sensitive *cdc7-1* cells can typically be synchronized at the G1-S boundary at 37 °C, yet failed to arrest at this temperature in a *cdc7-1 rif1∆* strain [16]. Furthermore, deletion of *RIF1* was found to increase the proportion of hyperphosphorylated Mcm4 in budding yeast whole cell extracts, as judged by slower mobility in sodium dodecyl sulfate polyacrylamide gel electrophoresis (SDS-PAGE) immunoblots [16,17,71]. To promote dephosphorylation, Rif1 possesses two conserved motifs for the docking of Glc7—the sole budding yeast protein phosphatase 1 [16,17,71]. Mutation of the Rif1 Glc7 docking domains was able to suppress *cdc7-4* and *dbf4-1* growth defects, consistent with it normally reversing the MCM phosphorylation carried out by DDK [17]. Further evidence for such a role was provided by the observation that a Rif1 Glc7 docking domain mutant resulted in increased Mcm4 phosphorylation, which could be reduced or prevented altogether in a *cdc7-1* background at permissive and non-permissive temperatures, respectively [71]. The idea of Rif1 targeting protein phosphatases to origin-bound MCM complexes is further supported by ChIP analysis carried out in both *S. cerevisiae* and the fission yeast *S. pombe*, which showed a reduction of Glc7 and *S. pombe* protein phosphatase 1 Sds21 and Dis2 at late-firing origins in the absence of Rif1 or with mutation of its protein phosphatase 1-binding motifs [71].

Intriguingly, a hint of another mechanistic layer in the opposing actions of Rif1 and Dbf4 has been provided by the key finding that Dbf4 can itself bind to Rif1 through the latter’s C terminus (amino acids 1790–1916) [16,17]. It is tempting to speculate that Rif1 may thus directly counteract DDK activity, however, the ability of DDK to phosphorylate Mcm4 in vitro was not inhibited by the addition of the purified Rif1 C terminus [16]. The inverse may also be true—namely, that DDK binding and potential phosphorylation of Rif1 hinders the latter’s ability to target Glc7 to origins. Indeed, putative conserved DDK and CDK phosphorylation sites are found adjacent to the protein phosphatase 1 docking domains in both *S. cerevisiae* and *S. pombe* Rif1. A *S. cerevisiae* Rif1 mutant for which nine of these serines were changed to alanine enhanced the temperature-sensitivity of *cdc7-1*, while changing them to aspartic acid as a phosphomimic had the opposite effect, reminiscent of what was observed when the docking sites themselves were mutated, and equivalent results were observed with similar *S. pombe* mutants [16,71].

Bringing things full circle, one further role of Rif1 is to potentially counteract DDK phosphorylation of Sld3. Although it has been clearly established that Sld3 is a crucial target for CDK phosphorylation, a significant phos-tag gel mobility shift has been observed for Sld3 in G1 phase *rif1∆* cells, consistent with phosphorylation, and this shift is prevented in *cdc7-4* cells at non-permissive (37 °C) temperature [17].

Interestingly, a recent report has uncovered a potential additional mechanism for DDK-mediated promotion of MCM phosphorylation [72]. SUMOylation of chromatin-bound Mcm2-6 subunits was detected, peaking in G1 phase after MCM loading, declining during S-phase, then rising again in M phase. Mcm7 showed a slightly different pattern, with SUMOylation persisting through most of S-phase, before declining at the end of S-phase. SUMOylation of Mcm6 was shown to increase its interaction with Glc7 [72], promoting the dephosphorylation of Mcm2-7 [16,17,71]. When DDK was inactivated, Mcm2-6 SUMOylation was no longer lost as cells transitioned from G1 to S-phase, suggesting that DDK mediates this process. As SUMOylated forms of Mcm4 did not appear to be phosphorylated by DDK, the authors speculated that DDK might instead act on deSUMOylation enzymes, although this remains to be investigated [72].

## 6. Targeting of DDK to Early Replicating Centromeric Origins of DNA Replication

Initiation events at budding yeast origins of DNA replication are temporally regulated, with individual origins characteristically firing in early, mid, or late S-phase [73]. DDK activity is limiting for DNA replication, as Dbf4 is present at low abundance and is required throughout S-phase to promote new initiation events [57]. DDK is therefore one of the determinants of which origins fire first in S-phase; however, only recently have some of the underlying mechanistic details been uncovered. Among the earliest origins to fire in S-phase are those associated with the 16 centromeric regions of *S. cerevisiae* chromosomes [73]. Similar findings have been obtained with other yeast species [74,75,76], *Trypanosoma brucei* [77], and *D. melanogaster* [78], suggesting that this is a conserved aspect of eukaryotic cell cycles. Interestingly, live cell imaging in *S. cerevisiae* has revealed that both Dbf4 and Cdc7 accumulate near spindle pole bodies and kinetochores in late M and early G1 phase [79]. The centromeric localization of Dbf4 was confirmed by ChIP-qPCR for cells arrested in G1 phase, but was strongly impaired when the genes encoding either chromosome transmission fidelity 19 (Ctf19) or chromomsome loss 4 (Chl4) (both kinetochore constituents) were deleted. This effect was specific for Dbf4 association at centromeres, as Dbf4 association with early-firing origins *ARS606* and *ARS607* was not altered in *ctf19∆* or *chl4∆* cells. The discovery that Dbf4 Myc-tagged at its C terminus is impaired for association with centromeres, but not with replication origins allowed researchers to determine that abrogation of DDK targeting results in a specific reduction in Sld3-Sld7 origin association and a delay in replication timing at centromeric regions [79]. Importantly, the recruitment of DDK to kinetochores also appears to promote sister chromatid cohesion by targeting the sister chromatid cohesion protein 2 (Scc2)-Scc4 cohesin loader to centromeres in G1 phase, which has also been observed in *Xenopus laevis* [80]. Thus, DDK likely plays a central role in coordinating S-phase onset with sister chromatid cohesion. Recently, Dbf4 localization at centromeres has also been observed in human cells, and DDK was implicated in regulating the recruitment of topoisomerase 2-alpha (TOP2A), which is required for chromosome condensation and sister chromatid separation [81]. Although the timing of Dbf4 centromere association was not coincident with the onset of DNA replication, this study involved the overexpression of tagged Dbf4. Thus, it would be interesting to observe if a stronger correlation is found with normal levels of Dbf4 expression.

## 7. Conclusions and Perspectives

To initiate DNA replication, DDK binds to and phosphorylates its essential target—the Mcm2-7 ring. This phosphorylation leads to gate opening between Mcm2 and Mcm5, allowing extrusion of single stranded DNA generated by origin melting. DDK also facilitates the association of one of the essential firing factors, Sld3, with origin DNA. A key feature of this DDK-dependent recruitment is that Sld3 interacts with DDK targets Mcm2, 4, and 6. Sld3 in turn targets Cdc45 to origins, thereby facilitating the formation of the CMG replicative helicase. As many of these mechanistic details have been determined through the use of in vitro systems, the additional construction of mutant strains will be required to confirm that they hold true in vivo. The recruitment of DDK to yeast centromeric sequences in G1 phase promotes early S-phase replication of these regions, and likely ensures proper coordination with sister chromatid cohesion through Scc2-Scc4 targeting to the same loci. Similar findings in other eukaryotes merit further investigation to establish the degree of mechanistic conservation.

Negative regulation of DNA replication by opposing DDK activity can occur via two distinct mechanisms. The checkpoint kinase Rad53 impedes DDK activity during S-phase replication stress. How Rad53 binds DDK to facilitate its phosphorylation has been characterized, exposing two FHA domain-mediated binding modes, one canonical and the other non-canonical. Rif1 and Mcm2-7 SUMOylation can each counteract DDK activity to prevent precocious DNA replication initiation in G1 phase by targeting Glc7 to dephosphorylate MCM subunits, yet exactly how Rif1 is itself recruited to origins of DNA replication, and the precise mechanism of Mcm2-7 SUMOylation, are open questions that remain to be investigated.
